# The man who mistook his neuropsychologist for a popstar: when configural processing fails in acquired prosopagnosia

**DOI:** 10.3389/fnhum.2015.00390

**Published:** 2015-07-17

**Authors:** Ashok Jansari, Scott Miller, Laura Pearce, Stephanie Cobb, Noam Sagiv, Adrian L. Williams, Jeremy J. Tree, J. Richard Hanley

**Affiliations:** ^1^Department of Psychology, Goldsmiths, University of LondonLondon, UK; ^2^School of Psychology, University of East LondonLondon, UK; ^3^Department of Life Sciences, Centre for Cognition and Neuroimaging, Brunel UniversityLondon, UK; ^4^Department of Psychology, College of Health and Human Sciences, Swansea UniversitySwansea, UK; ^5^Department of Psychology, University of EssexColchester, UK

**Keywords:** face-recognition, FRUs, Navon, mental-imagery, prosopagnosia, featural processing, holistic processing, configural processing

## Abstract

We report the case of an individual with acquired prosopagnosia who experiences extreme difficulties in recognizing familiar faces in everyday life despite excellent object recognition skills. Formal testing indicates that he is also severely impaired at remembering pre-experimentally unfamiliar faces and that he takes an extremely long time to identify famous faces and to match unfamiliar faces. Nevertheless, he performs as accurately and quickly as controls at identifying inverted familiar and unfamiliar faces and can recognize famous faces from their external features. He also performs as accurately as controls at recognizing famous faces when fracturing conceals the configural information in the face. He shows evidence of impaired global processing but normal local processing of Navon figures. This case appears to reflect the clearest example yet of an acquired prosopagnosic patient whose familiar face recognition deficit is caused by a severe configural processing deficit in the absence of any problems in featural processing. These preserved featural skills together with apparently intact visual imagery for faces allow him to identify a surprisingly large number of famous faces when unlimited time is available. The theoretical implications of this pattern of performance for understanding the nature of acquired prosopagnosia are discussed.

## Introduction

Several acquired prosopagnosic patients have been reported with severe difficulties in identifying faces despite being able to recognize other classes of objects (e.g., McNeil and Warrington, 1993; Riddoch et al., 2008; Rivest et al., 2009; Rossion et al., 2011). The existence of such cases can be used to suggest that a special system dedicated to faces that is not involved in object recognition has been damaged. However, because human faces share the same basic features, it could also be argued that faces are simply very “difficult objects” to recognize. Partial damage to the recognition system might affect faces, but not objects if faces require an additional level of visual processing relative to objects. This position is weakened, however, by the case of acquired object agnosic CK (Moscovitch et al., 1997) and the case of developmental agnosic AW (Germine et al., 2011) who are able to identify faces despite having significant difficulties in recognizing everyday objects.

Farah (1990, 1991, 2000) has claimed that recognition of objects and faces typically rely on two distinct forms of visual processing. In Farah's exposition, objects typically require decomposition into parts before they can be identified. For example, identifying a chair might involve recognizing that it has some legs, a flat surface on top of these legs and some sort of back section. The ability to interpret and encode individual parts will be referred to here as “featural processing.” Conversely, Farah claimed that faces cannot be recognized by decomposition into parts and are therefore recognized almost exclusively using another system that sees the whole. The ability to combine individual parts into a whole has been given various names by different authors such as holistic, gestalt, or configural processing (e.g., Calis et al., 1984; Diamond and Carey, 1986; Young et al., 1987; Moscovitch et al., 1997; Barton, 2009; see Maurer et al., 2002 for a review). In this paper, following Maurer et al. (2002) we will use holistic to refer to the ability to “glue” individual elements of a face into a coherent whole and configural to refer to “first-order relations that define faces (i.e., two eyes above a nose and mouth)” (p. 255).

Can the dissociation between object agnosia and prosopagnosia be explained *solely* in terms of the distinction between featural and configural processing? There is strong evidence that the agnosic patient CK (Moscovitch et al., 1997; Moscovitch and Moscovitch, 2000) has preserved configural processing despite impaired featural processing. CK could recognize familiar faces whenever the configural information appeared to be accessible from the visual stimulus. He therefore performed well-when faces were presented as cartoons, as caricatures, in disguise, and when a single internal feature had been removed. He could also recognize famous faces when all of the external features had been removed, and when they were vertically misaligned. Crucially, however, CK was severely impaired at recognizing inverted famous faces where the configural information is hard to extract. He also performed poorly on other facial recognition tasks in which the configural information was reduced or absent such as recognition of famous faces from their external features and recognition of horizontally misaligned famous faces. Although normal controls were inconvenienced by these manipulations, they all performed very much better than CK. Presumably this is because their object recognition system (unlike that of CK) is able to perform some degree of compensatory feature-based processing on a face when configural information cannot be accessed. On the basis of CK's preserved and impaired pattern of performance with faces, Moscovitch et al. (1997, p. 592) concluded that the ability to identify faces depends crucially on the “spatial relations of the internal features of a face (the eyes, the nose, and the mouth) to each other” and is quite separate from the ability to recognize objects.

Important evidence concerning the existence of a configural deficit in prosopagnosia has come from the study of patient PS who has no low level visual processing impairments. PS is able to distinguish Arcimboldo faces and Mooney faces (Rossion et al., 2011) from non-facial stimuli accurately and at normal speed. This finding suggests that she has preserved ability to process faces holistically. However, PS is severely impaired at matching upright unfamiliar faces (Busigny and Rossion, 2010), but performs as accurately and quickly as controls at matching inverted unfamiliar faces. This dissociation suggests that she can use featural but not configural information to distinguish one face from another. Further evidence of a configural deficit is that PS shows no evidence of perceiving facial features or facial composites more accurately when they appear in the context of a whole face (Ramon et al., 2010) than when they appear alone. This finding suggests that she processes individual facial features independently of the overall facial configuration. Another acquired prosopagnosic (GG) also showed no face inversion effect, no face composite effect and no part-whole advantage on unfamiliar face recognition tasks, consistent with impaired configural but preserved featural processing (Busigny et al., 2010). Barton et al. (2003) reported an acquired prosopagnosic who could detect facial feature changes but was relatively insensitive to manipulations that distorted overall facial geometry of unfamiliar faces. Similar results have also been reported by de Gelder and colleagues (e.g., Huis In ‘t Veld et al., 2012) with individuals who have the developmental variant of prosopagnosia.

Such studies provide convincing evidence that the problems that prosopagnosic patients such as PS and GG experience in processing *unfamiliar* faces are associated with a configural processing deficit. Nevertheless, there is less evidence that the core deficit in recognizing *familiar* faces in acquired prosopagnosia is caused by a configural processing impairment. It has never been demonstrated that prosopagnosic patients perform well at identifying familiar faces when featural rather than configural processing appears to be critical for recognition. For example, PS was impaired relative to controls at recognizing the inverted faces of the students that she taught (Busigny and Rossion, 2010). Similar findings in another individual with acquired prosopagnosia were reported by Rivest et al. (2009), who performed much worse than controls at identifying familiar faces even when they were inverted or fractured.

Such findings raise the possibility that the core deficit in recognizing familiar faces in at least some forms of prosopagnosia is at a deeper level than a purely configural processing deficit. For example, Burton et al. (1991) and Burton and Young (1999) argue that associative prosopagnosics have an impairment at the level of face recognition units (FRUs). More precisely, they claim that the appropriate FRU may be activated when a familiar face is seen, but the connections between the FRU and stored knowledge about the person are so weak that the face is not overtly recognized. On the assumption that the same FRUs are used to recognize familiar faces regardless of orientation, an impairment of this kind should affect recognition of familiar faces regardless of whether they are presented upright, inverted or fractured. The view that the familiar face identification impairment in prosopagnosia is caused by a configural processing deficit would therefore be bolstered if a patient can be found whose performance when recognizing *familiar* faces shows preserved featural but impaired configural processing. Despite performing badly on tests with unfamiliar faces that require configural processing, such a patient might perform well at familiar face processing tasks such as inverted or fractured face recognition. Below we report the case of an individual (DY) who appears to fit this profile. As we will demonstrate, however, his performance is different in some interesting respects from that typically found in acquired prosopagnosia.

A total of nine studies are presented, two demonstrating the specificity of DY's prosopagnosia, four manipulating levels of configural and featural processing of faces, one manipulating global vs. local processing of non-face stimuli and two investigating visual imagery for famous faces. Experiment 1 addresses DY's ability to make within-category discriminations for non-face visual objects, while Experiment 2 assesses his performance on a visual recognition task of objects and famous faces that have been matched for difficulty. Experiment 3 uses the classic face inversion study to demonstrate the impact of this paradigm on DY's processing of unfamiliar faces while Experiment 5 addresses the impact of inverting a small set of famous faces that he sometimes recognizes. Experiments 4 and 6 use variants of paradigms devised by Moscovitch et al. to pit featural and configural processing against one another using familiar faces. Experiment 7 uses a classic cognitive paradigm devised by Navon (1977) to investigate global and featural processing in non-facial stimuli. This experiment provides evidence that DY's configural deficit is of a general kind and is not confined to the processing of facial materials. Finally, Experiment 8 adapts the Young et al. (1994) approach to looking at mental imagery for faces in the patient using a forced-choice recognition task while Experiment 9 addresses this issue using a free recall paradigm.

## Case history

**Figure 1:**
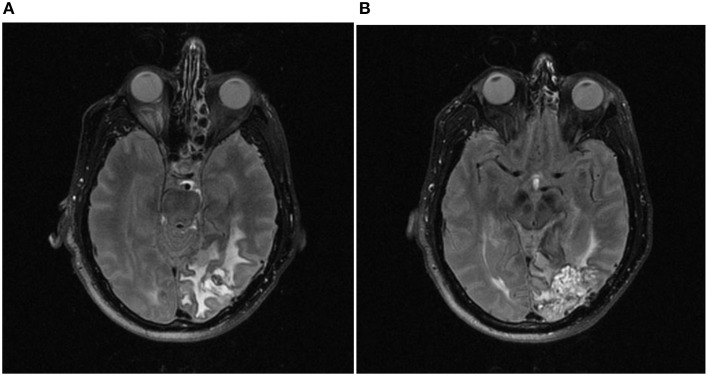
**Two axial T2 FLAIR MR images showing the location of DY's lesion (1 × 1 mm in-plane resolution, 3 mm slice thickness; slice locations relative to the nasion are as follows: (A) −35 mm, (B) −26 mm)**. The lesion is confined mainly to the posterior regions of the right hemisphere (predominantly occipital, but also including the precuneus), and extending ventrally into temporal lobe regions.

DY is a right-handed male sales executive born in 1946. After a routine eye check in 1999, a left homonymous hemianopia was revealed and a subsequent MRI scan identified a large arterio-venous malformation (AVM) located in the right posterior hemisphere. In 2000, DY was treated with embolization of the AVM which involves obstruction of the AVM blood vessels with a special glue. This was followed by gamma-knife surgery which captured 90% of the malformation. This is a procedure for treating tumors and AVMs using gamma radiation delivered to a precise location by concentrating multiple beams from weaker sources. In 2001, DY suffered a right occipital intracerebral hemorrhage resulting from bleeding from the AVM. Figure 1 presents two T2-weighted fluid attenuated inversion recovery (FLAIR) images taken 10 years later in 2011 illustrating DY's lesion. The MR signal from the CSF is suppressed and results in the lesion being more prominent. DY's lesion appears to be confined to the posterior RH, but affecting parietal, temporal, and a large part of the occipital lobe. Regions affected include the precuneus, the cuneus, and lingual gyrus. Also affected are middle temporal and fusiform gyri in the temporal lobe, and the middle and inferior occipital gyri. The regions affected include Brodmann areas 7, 17–19, and 37.

DY recovered in hospital and reported significant cognitive difficulties, mostly memory problems and disorientation. DY felt very distressed because he did not recognize his wife or grandchildren when they came to visit him in hospital. After leaving hospital, DY reported several cognitive difficulties. Initially he experienced confusion in certain situations such as looking at items in the fridge, and he found looking at the shelves in supermarkets unbearable. DY described the experiences, in his own words as “dyslexia of the eyes.” Most of these initial difficulties resolved, but his ability to recognize faces has never returned to normal. DY recalls an incident when shopping with his wife, where they separated. Later, upon seeing him again in the street, his wife walked toward him and waited to be acknowledged; he did not recognize her and walked straight past. He has now adopted techniques such as remembering his wife's clothes if they go out so that he can tell who she is when in crowds. DY reports that he is often able to perform relatively well in everyday life with his impairment because if he is expecting to see somebody in a location he is more likely to recognize them successfully by using memory of voices or clothing. In fact, using his very strong visual memory skills, he is able to disguise an extremely profound impairment.

During preliminary testing, analysis of DY's verbal protocol while naming faces suggested that the majority of his correct recognitions were based on individual features found in faces rather than a simple recognition of the face. During this phase of testing, he named a picture of his neuropsychologist (and first author, AJ) as the popstar George Michael. Whilst incorrect, this showed that DY was using AJ's goatee beard, slightly darker skin and gold earring to arrive at the name of George Michael. Now that DY associates the goatee beard with AJ, he has commented a number of times that if AJ shaves off the goatee beard, he will no longer be able to “recognize” him.

DY currently reports no noticeable difficulties with recognizing objects but does have difficulty in finding his way, and often has to ask for directions. There were no indications of difficulties with reading or with color recognition. There was no evidence of a loss of long term memory. The studies reported were conducted over a period of 5 years starting when DY was 60 years of age and in generally good health.

## Participants

DY's performance was assessed relative to either published norms or against a group of age and WTAR-IQ (Wechsler, 2001) matched healthy male control participants. Due to the long time-span of the studies reported, different sets of controls were used for each study. Crawford and Garthwaite's (2002) method for comparing a single case with a group of control subjects was used for statistical comparison of DY's performance against that of the controls.

## Ethical approval

All the studies described received approval from the University of East London's Ethics Committee.

## Background testing/standard cognitive functioning

### Visuo-spatial processing

#### Visual object and space perception battery (VOSP; Warrington and James, 1991)

DY was normal on five out of the eight subtests of the VOSP (Table 1) and his main impairments were on the object identification tasks of “silhouettes” and “progressive silhouettes.” In these tests, only the outline forms of the objects are visible. Poor performance on all of these tests suggests that DY suffers from difficulties with recognition of the outlines of shapes, termed, *global* forms. In object decision tasks when the *parts* of the objects are available, performance is at a normal level.

**Table 1:** **Breakdown of DY's performance on VOSP and BORB subtests**.

**Test**	**Sub-test**	**DY's performance**	**Interpretation**
VOSP	Incomplete letters	19/20	Normal
	Silhouettes	14/30	Impaired
	Object decision	16/20	Normal
	Progressive silhouettes	11/20	Impaired
	Dot counting	10/10	Normal
	Position discrimination	15/20	Impaired
	Number location	8/10	Normal
	Cube analysis	10/10	Normal
BORB	Copying simple shapes	Accurate copying	Normal
	Length match (Horizontal)	20/30	Impaired
	Length match (Vertical)	26/30	Normal
	Size match	26/30	Normal
	Orientation match	25/30	Normal
	Gap match	37/40	Normal
	Overlapping figures	Paired letters (1.0:1.2)	Normal
		Triple letters (1.0:12)	Impaired
		Paired geometric shapes (1.0:1.1)	Impaired
		Triple geometric shapes (1.0:1.2)	Impaired
		Line Drawings (1.0:1.4)	Impaired
	Minimal feature match	25/25	Normal
	Foreshortened view	25/25	Normal
	Object decision	114/128	Normal

#### Birmingham object recognition battery (BORB; Riddoch and Humphreys, 1993)

On this extensive test of visuo-spatial abilities which addresses different levels of visual processing, DY's performance was within the normal range on all tests apart from overlapping figures and matching horizontal lines (Table 1). He was able to recognize individual letters, geometric shapes, and line drawings with no difficulty; however, when these were overlapped with each other, DY's performance time in correctly naming the figures fell outside the normal range.

#### Facial expressions of emotions: stimuli and tests (FEEST; Young et al., 2002)

In this test of ability to process emotions from faces, a series of faces showing six standard emotions are presented on a computer screen with six verbal labels corresponding to each emotion. Apart from particular problems in identifying the “anger” emotion, DY's performance was within one standard deviation of the control mean and was often superior.

#### Recognizing mooney faces (Busigny et al., 2010)

Mooney faces are two-tone black and white pictures of faces that do not contain clear facial features. It is difficult to see them as faces when they are presented as inverted. This finding suggests that holistic processing is required in order to identify Mooney faces accurately.

The procedure developed by Busigny et al. (2010) was used with DY. Busigny et al.'s procedure involved presentation of 80 black and white Mooney faces selected from an original set created by Schurger and colleagues (Art of Science Competition, Princeton University, http://www.princeton.edu/artofscience/gallery). The 80 stimuli were presented both upright and upside-down randomly in two blocks of 80 trials. Each stimulus was presented on a gray background and the participant had to decide whether or not they saw a face by pressing one of two keys on the computer keyboard; they were informed that they should only use the “face” response if they saw a face upright and that anything else should be categorized as a non-face. Participants were instructed to respond as accurately and as quickly as possible. Following their response, a central fixation cross was presented for 300 ms and then a gray screen for 300 ms before the next stimulus.

The results showed that DY was correct on 127/160 correct which was significantly different from that of his matched controls (*M* = 146.4, *SD* = 7.2), *t*_(7)_ = 2.54, *p* = 0.019. DY's average response time per trial was 1317 ms which was also significantly different to that of the controls (*M* = 940 ms; *SD* = 117), *t*_(7)_ = 3.04, *p* = 0.009. It therefore appears that DY, has an impairment in holistic processing of unfamiliar faces.

#### Benton face-matching test (Benton and van Allen, 1968)

On the Benton Face-Matching Test, DY's score of 41 placed him just within normal limits. At face value, this result could be interpreted as normal face perception. Indeed, De Renzi and Pellegrino's (1998) case Anna, similar to DY, did not exhibit object agnosia and showed poor performance on a range of face-perception tasks. De Renzi et al. (1991) took Anna's normal performance on the Benton test as implying intact face perception. However, Duchaine and Nakayama (2004) have shown that this test has poor specificity for picking up face recognition difficulties. Further, Farah (1991) rightly cautions against using just accuracy for interpreting performance on this task since this can mask an abnormal strategy (an issue that is very relevant to DY's performance in Experiment 6 of the current study—see later). As pointed out by Newcombe (1979, p. 319) “Some prosopagnosic patients are reported to match faces normally… Latencies, however, are not invariably measured.” To address this issue, DY's performance on the test was timed and it was found that he took 12 min to complete the task. This is an extremely slow time and DY (who tends to verbalize his thoughts when performing such tasks) laboriously compared different features to arrive at his seemingly “normal” accuracy score. The conclusion is therefore that DY's overall processing of the unfamiliar faces on this task is abnormal. This suggests finds it difficult to use configural information to distinguish one unfamiliar face from another.

## Experiment 1: within-category naming

In order to evaluate the specificity of DY's visual recognition abilities, his *within-category* naming was assessed.

### Stimuli and procedure

Participants were shown a series of 20 images of familiar objects in each of four categories (national flags, types of car, famous buildings, and football shirts) and were asked to name each exemplar. All exemplars of flags, with a few exceptions, conform to the same rectangular shape and therefore the only way to name the country is by processing the specific information within the flag. Similarly, cars tend to conform to a prototypical shape but differ along dimensions such as relative sizes of different parts, insignias, etc. The exemplars of buildings were chosen so that there were visually similar exemplars such as famous bridges. Finally given DY's interest in football, shirts belonging to teams in the English Premier League were used; as in the case of flags, all football shirts have the same shape and so identity of the particular football club needs to be done by analysis of each exemplar's colors and insignias.

### Participants

DY's responses were compared to those of eight healthy male controls matched for age (range 55–65 years, mean 60.9 years) and education.

### Results and discussion

**Figure 2:**
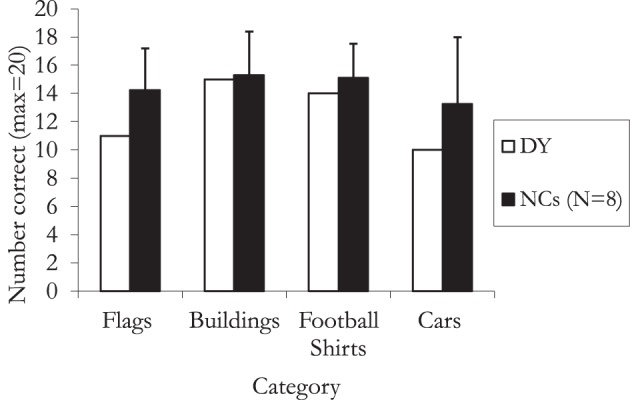
**Within-category naming ability (error bars represent one standard deviation)**.

Figure 2 shows DY's performance compared to that of the controls and shows that he was within one standard deviation or less of the control mean and therefore within normal limits (all *p* > 0.05). This finding demonstrates that DY does not show a within-category recognition deficit, implying that his naming difficulties are restricted to faces.

## Experiment 2: familiar object and face recognition

One criticism that can be leveled against using intact object recognition in the context of face recognition difficulties to suggest a specific impairment in the latter ability is that normal controls are likely to perform near ceiling levels on both recognition tasks involving faces and everyday objects (Farah, 1994). Judging a neuropsychological patient's performance as ‘intact’ relative to such ceiling effects therefore can be questionable. To overcome this potential criticism, DY was administered a naming test in which the difficulty of faces and objects had been titrated to be of equal difficulty.

### Stimuli and procedure

The Essex-Exeter Matched Difficulty Object and Faces tests (Lyons et al., 2002) test has been specifically created to include sets of objects and faces that have been matched for naming difficulty such that normal performance on neither test is at ceiling levels. The test consists of four subsections, two for faces and two for objects. Each of the subsections contains 31 items resulting in a total of 62 items being presented for each category. Stimuli are presented on a computer screen for an unlimited time with the participant having to provide the name or sufficient semantic information to demonstrate recognition. DY's responses were compared to those of the mean and standard deviation for the 50 participants in the original Lyons et al. (2002) paper.

### Results and discussion

DY named 35/62 of the objects (*M* = 41.2, *SD* = 6.5) showing that even when items have got quite specific names (e.g., puffin) he performs within the control range, *t*_(49)_ = 0.94, *p* > 0.05. His responses were both accurate and fast. DY achieved a score of 33/62 (*M* = 42.2, *SD* = 12.6) for face naming, which is also within the normal range, *t*_(49)_ = 0.72, *p* > 0.05. This performance seems paradoxical for an individual who claims not to be able to recognize his wife and other close family members. However, as with his performance on the Benton test, it is important to take account of the method that he used to achieve such a level of performance. Unlike his rapid responses to objects, DYs responses to faces were slow and faltering. Analysis of his verbal protocol while naming faces suggested that the majority of his correct responses were based on individual features found in faces rather than a “normal” recognition of the face^1^. For example, when shown an iconic picture of Marilyn Monroe, it took DY 7 s to arrive at a name and then said that it was a guess based on her beauty spot and the shape of her lips! Also, rather than stating who the person was, he asks the question “Is that Marilyn Monroe?” In sum, although sometimes DY is able to recognize faces, his method of doing so is far from normal and we believe that the apparently normal accuracy score for naming faces masks a profound face recognition difficulty. In Experiment 4, we will demonstrate formally this impairment by measuring RT as well as accuracy when investigating DY's ability to recognize famous faces. Experiment 3 examines learning of unfamiliar faces because featural cues to identity are much less likely to be available to DY with unfamiliar than with famous faces.

## Experiment 3: cambridge face memory test (Duchaine and Nakayama, 2006)

The Duchaine and Nakayama (2006) Cambridge Face Memory Test provides an opportunity to investigate whether DY is significantly impaired at learning new faces. It is designed to explore recognition memory for unfamiliar faces in both upright and inverted conditions. The standard finding from normal controls is superior memory for faces when seen upright compared to when seen inverted. This “face inversion effect” has been used as a hallmark indication of the special nature of face processing as under normal circumstances, faces are processed as a configural whole. However, when faces are inverted, this dedicated form of processing is disrupted, increasing the reliance on featural processing. If DY's normal accuracy for familiar faces in Experiment 2 is associated with excellent featural processing and impaired configural processing, it would follow that DY should show a greatly reduced face inversion effect relative to controls.

### Stimuli and procedure

Duchaine and Nakayama's standard procedure was employed. Briefly, participants are presented with black and white images of unfamiliar faces to memorize. Immediately after a set of learning trials for each face or set of faces, the participant is asked to select the target from among an array that includes two distractors. There are three stages increasing in difficulty with a different number of stimuli for each section: Introductory (*N* = 18), Novel (*N* = 30), Novel + Noise (*N* = 24). The test was completed by DY and each normal control participant in an upright condition followed by the inverted condition. (It should be noted that while inverting a face could involve disruption of configural processing, the CFMT also introduces an additional memory component because there is a delay between initial learning of the to-be-recognized upright face and the test trials with inverted faces. Therefore, we acknowledge that there is a contamination of a pure inversion effect as measured by the CFMT. However, since many other research groups have used this measure, we do so while acknowledging this caveat).

### Participants

DY's performance was compared to that of 10 normal controls matched for age and WTAR IQ. The controls had a mean age of 59.2 (range 51–67) and mean IQ of 104.7 (range 92–115).

### Results

Table 2 presents the performance of DY and the NCs as a function of condition, broken down by sub-category within condition. As expected, collapsing across the different conditions, the NCs show a superiority for recognizing faces upright compared to inverted, *t*_(9)_ = 6.47, *p* < 0.001. Conversely, DY performs at least as well on the inverted faces as on the upright faces. Overall, DY was significantly impaired relative to controls in the upright condition, *t*_(9)_ = 3.18, *p* = 0.01, but within normal limits for the inverted condition, *t*_(9)_ = 0.74, *p* = 0.48. Looking more closely at the sub-categories, there was a significant difference between DY and the normal controls in the upright introductory, *t*_(9)_ = 11.32, *p* < 0.001 and novel sub-categories, *t*_(9)_ = 2.49, *p* < 0.05. Contrasting with the upright condition, DY was always within normal limits in the inverted condition (all *p* > 0.05). There was no significant difference between DY and the controls in the most difficult sub-category of both conditions but as can be seen, his performance in both cases was below chance. Only in the condition where noise is added (a manipulation that disrupts local/feature processing more than global processing) did DY show any evidence of poor performance.

**Table 2:** **Performance on Cambridge Face Memory Test (standard deviations in parentheses)**.

**Condition**		**DY**	**NCs (*N* = 10)**
Upright	Introductory (max = 18)	12	17.7 (0.5)
	Novel (max = 30)	7	17.6 (4.1)
	Novel + Noise (max = 24)	7	11.6 (3.5)
Inverted	Introductory (max = 18)	11	12.9 (4.2)
	Novel (max = 30)	14	12.9 (2.0)
	Novel + Noise (max = 24)	6	9.4 (2.5)

Directly comparing the difference between the upright and inverted conditions, using Crawford and Garthwaite's (2005) Revised Standardized Difference Test (RSDT), it was found that DY was significantly different to NCs, *t*_(9)_ = 10.45, *p* = 0.00004.

### Discussion

DY's poor performance in the upright condition clearly reveals a significant impairment in learning new upright faces. Interestingly, DY showed no significant impairment relative to controls in the inverted faces condition consistent with the view that his featural processing of faces is normal. The results strongly suggest that he is relying on featural rather than configural information to identify previously unfamiliar faces. As expected, the normal controls display the expected upright superiority effect achieving higher scores in the upright condition than the inverted condition. However, DY did not show the upright superiority effect and in fact performed slightly better in the inverted condition than the upright condition (Table 2). The finding that prosopagnosics perform at least as well on inverted as upright faces has been termed the “inverted inversion effect” (Farah et al., 1995) and is even found in some cases of developmental prosopagnosia (Duchaine et al., 2006; Le Grand et al., 2006; Bate et al., 2008).

The absence of an inversion effect for DY must be treated with some caution, however. First, the performances of DY and controls were near floor in some of the inverted conditions. Second, because of the structure of the CFMT, it is not possible to counterbalance half sets of the upright and inverted conditions. Since our primary aim was to objectively demonstrate DY's difficulty in remembering pre-experimentally unfamiliar faces, we conducted the upright condition first and followed this with the inverted condition in order to test for the inversion effect. So one explanation for the lack of inversion effect in DY is that it may have come about because the inverted faces were presented after the faces had already been presented in the upright condition.

## Experiment 4: “face-fracturing” test

In this experiment, we investigated the time that it takes for DY to recognize a famous face. It seems highly likely that his recognition strategy will lead to extremely long RTs even if it sometimes produces accurate performance. We tried to ensure fairly accurate performance by using a set of faces that DY was able to identify consistently. The stimuli were generated by asking DY's wife to provide a list of names of famous people who, she felt DY recognized on a consistent basis when they appeared on TV or in the newspapers. It was stressed that this recognition should be based on visual attributes rather than their names, voices or any semantic information. Using a variety of sources, 25 easily recognizable photos were compiled for the set of stimuli. The critical dependent variable was the speed with which these faces could be identified by DY.

A second goal of the experiment was to investigate DY's ability to recognize fractured faces. Moscovitch et al. (1997) showed that their object agnosic patient CK had impaired recognition of faces that were created by taking intact photographs and cutting them into five or six parts. Individual features (eyes, noses, etc.) were kept intact and the first-order relations between the features were kept intact (e.g., the eyes were kept above the nose which was kept above the mouth, etc.). Moscovitch et al. found that whilst CK's performance was completely normal in the intact condition, his performance fell six standard deviations below the mean of the controls when the same faces were “fractured.” Since exactly the same visual information was available in both conditions, these data strongly suggest that the manipulation of isolating features spatially by destroying the gestalt impaired CK's recognition ability. In Experiment 4, Moscovitch et al.'s paradigm was adopted for use with DY.

### Stimuli

**Figure 3:**
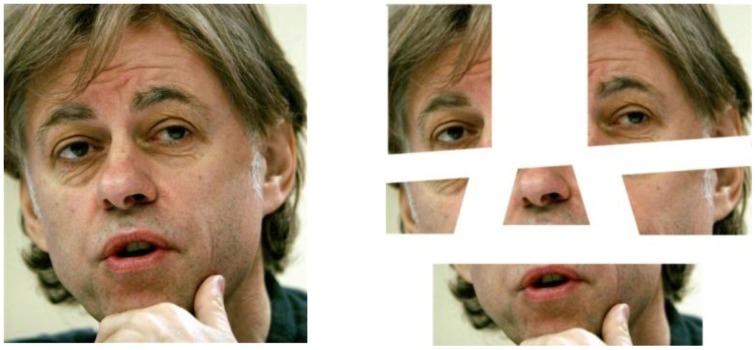
**Examples of an Intact and Fractured face (Bob Geldof)**.

Photos that DY's wife thought he would recognize were compiled for the ‘intact’ set of stimuli. It was stipulated that all the faces had to be of individuals who had come to public prominence before 1999 when DY was first diagnosed with brain damage. Then using image-manipulation software (Corel Draw), each of these color photos was digitally cut using the criteria suggested by Moscovitch et al. (1997). Figure 3 gives an example of the face of Bob Geldof in the two conditions. In total 25 faces were used.

### Participants

DY's performance was compared to a group of six male control participants who also participated in Experiment 1.

### Procedure

Each list of intact and fractured faces was divided into two equal sets to allow counter-balancing of conditions. Half of the intact set and half of the fractured set were used on Day 1 of testing and then the remainder were used on Day 2 of testing which took place a week later. Stimuli were presented individually on a laptop using E-prime software and the participant was asked to name as quickly as possible the individual in the display. This allowed accuracy and response times for correctly named stimuli to be measured.

### Results

In terms of naming accuracy, controls named 24/25 of the intact faces (*SD* = 0.63) and 23.17/25 of the fractured faces (*SD* = 1.17). DY performed very similarly to the controls naming 23 faces in the intact condition, *t*_(5)_ = 1.46, *p* > 0.05, and 22 in the fractured condition, *t*_(5)_ = 0.93, *p* > 0.05. DY's accurate performance for intact faces is expected given that the stimulus set was created by asking his wife for faces that he consistently recognizes; however, it is striking that fracturing has no significant impact on his overall accuracy. By contrast, CK (Moscovitch et al., 1997) was severely impaired by a fracturing manipulation.

**Figure 4:**
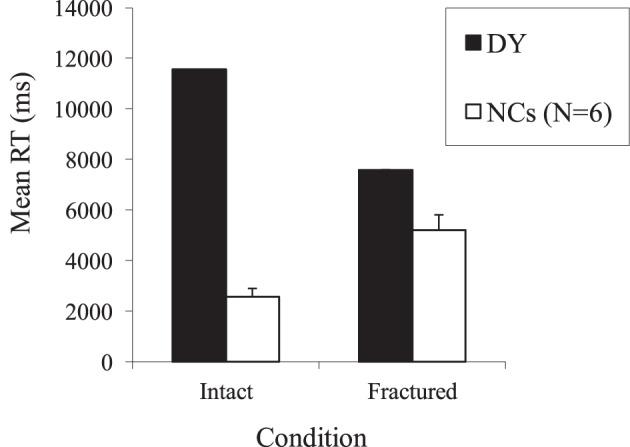
**Mean response times for DY and matched controls (NCs) on the Moscovitch et al. (1997) face-fracturing paradigm**.

To investigate performance further, the average times for correct responses were compared (see Figure 4). As can be seen, the normal pattern of performance is that the fractured condition takes longer, almost double that of the intact condition. However, DY shows the opposite pattern with his average time in the fractured condition being only 2 s slower than that of the controls whereas his average time for the intact condition was on average 10 s slower. Directly comparing the difference between RTs in the intact and fractured conditions (Crawford and Garthwaite, 2005), revealed that DY was significantly different from NCs [*t*_(5)_ = 10.66, *p* = 0.00013].

### Discussion

The results demonstrated that DY takes a relatively long time to identify familiar faces despite his accurate performance. They also revealed that face fracturing has no impact on his familiar face identification accuracy. Furthermore, the time that DY took to arrive at an answer was in fact faster in the fractured condition, and, anecdotally, he reported that he found this condition easier. Overall, DY's performance implies reliance on featural processing irrespective of whether the face is presented intact or fractured. It may well be that he performs more quickly in the fractured face condition because his impaired configural processing skills interfere with face recognition in the standard condition (cf. Farah et al., 1995; Boutsen and Humphreys, 2002). The finding that his performance was faster in the fractured condition is consistent at some levels with the evidence of inversion superiority in Experiment 3. As suggested by Farah et al. (1995, p. 2093), this “concept of dominance by a specialized but impaired brain system” has been invoked to explain the discrepancy found in other areas of neuropsychology such as linguistic performance following left-hemisphere brain damage. There may be no interference in the fractured condition because such stimuli do not activate DY's impaired configural processing system.

## Experiment 5: inverted famous faces

The results of Experiments 3 and 4 suggest that DY's problems in identifying faces are associated with a deficit in configural processing despite normal featural processing. Experiment 5 investigated his ability to identify inverted pre-morbidly familiar faces. If he uses featural rather than configural information, it would be predicted that he would show no effect of inversion on accuracy of naming or on the time necessary to recognize a face as being familiar.

### Stimuli and procedure

In the first phase of this experiment, the faces from Experiment 4 were used again. They were inverted and presented on a laptop computer. There was no time limit. Following Moscovitch and Moscovitch (2000), an answer was deemed correct if the name was provided or if sufficient semantic information to demonstrate recognition was produced. At least a week separated the presentation of the upright and inverted faces. In the second phase of the experiment, which took place several months later, new inverted pictures of the 25 faces used in Experiment 4 were presented. We used new pictures of the celebrities to avoid any possible priming from having seen the images used in Experiment 4. Participants had to respond with a key press as to whether or not they recognized the inverted face as familiar, a procedure that allowed RT for recognition to be measured.

### Participants

DY's performance was compared to that of nine normal controls matched for age and WTAR IQ. The controls had a mean age of 63.1 years (range 59–69) and mean IQ of 112 (range 90–117).

### Results

Table 3 shows performance of DY and the matched controls for recognition in the inverted and upright conditions. DY's Phase 1 upright accuracy scores come from Experiment 4. There was no significant difference between DY and the control participants in the inverted condition, *t*_(8)_ = 0.76, *p* > 0.05, with his performance falling within one standard deviation of the control mean. The accuracy with which inverted faces were recognized in Phase 2 was also within the normal range, as was the length of time required to make these identification decisions.

**Table 3:** **Identification of upright and inverted famous faces (max = 25; standard deviations in parentheses)**.

	**DY**	**NCs (*N* = 9)**
**PHASE 1**		
Upright	23	24.6 (1.14)
Inverted	9	12.4 (7.78)
**PHASE 2**		
Inverted accuracy	10	13.1 (5.0)
Inverted RT (ms)	3586	3120 (1054)

### Discussion

When faces that DY can recognize are inverted, his recognition is within normal limits in terms of both speed and accuracy. It is also interesting to note that, like the normal controls, DY's performance was much better in the upright than in the inverted condition. A strong version of a theory that suggests that DY only has access to featural processing might predict that his performance should be the same upright and inverted since he would be basing his recognition on a simple featural match. One possibility, therefore, is that the recognition of individual facial features is, to at least some extent, orientation specific (Moscovitch and Moscovitch, 2000). If so, inversion will make not only configural processing but also featural processing of faces more difficult. Consistent with such an account, a number of experiments have shown that inversion disrupts face feature perception in matching tasks (e.g., Yovel and Kanwisher, 2004; Yovel and Duchaine, 2006). If it is assumed that face fracturing does not interfere with featural processing to the same degree as inversion, then this would explain why performance was much better for fractured faces in Experiment 4 than for inverted faces in the current experiment.

## Experiment 6: external features

Moscovitch and Moscovitch (2000) argued that if their patient CK's object agnosia was driven by reliance on a face-processing system that is based on configural processing as a result of damage to the part-based system, he would suffer if the main configural information is removed from a face. To explore this, they created stimuli in which the main configuration of eyes, nose and mouth were cut and were replaced by a white space. They found that whereas healthy controls were somewhat impaired by this manipulation (with recognition dropping to 63.8% of that with the faces whole), CK was grossly impaired with his performance dropping to 33.3%. Experiment 6 was conducted to investigate DY's recognition of familiar faces using only external features.

### Stimuli and procedure

**Figure 5:**
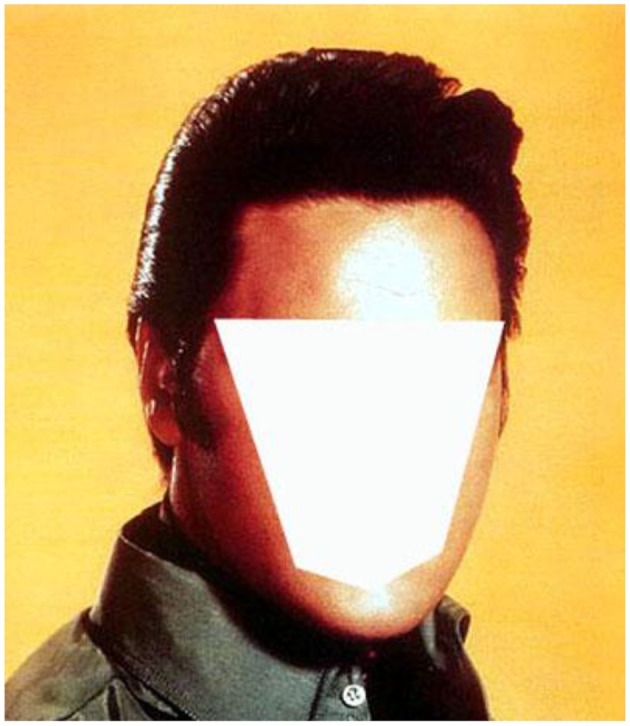
**Example of a stimulus from Experiment 6 with the internal features of Elvis Presley's face digitally removed**.

A corpus of 42 famous faces was assembled. Some of these were of faces that DY is known to recognize and had been used in Experiments 4 and 5; however, care was taken to make sure that the same photograph was not used for the current study and instead new photographs were found. The remaining faces were of famous individuals who are often recognized from their very particular hairstyles or other features outside the face. Following, Moscovitch and Moscovitch's (2000) procedure, a line was drawn just above the eyes and the edges of this were joined to points just either side of the mouth and finally these two points were brought together just underneath the mouth. The space created by these five lines was filled in with white space (see Figure 5). All participants were presented the stimuli in the same order on a laptop computer. There was no time limit and, again, an answer was deemed correct if the name was provided or sufficient semantic information to demonstrate recognition was produced.

### Participants

DY's performance was compared to that of the nine controls who took part in Experiment 5.

### Results

DY identified 31/42 of the faces correctly compared to the mean of the controls which was 32.1 (*SD* = 6.37). There was no significant difference between these scores, *t*_(8)_ = 0.17, n.s.

### Discussion

DY's unimpaired performance on this test shows that he is able to recognize familiar faces from their external features. This finding represents a dissociation with patient CK (Moscovitch and Moscovitch, 2000) whose performance on this task was severely impaired. It provides further evidence that DY's familiar face recognition impairment is characterized by normal featural processing but impaired configural processing.

## Experiment 7: navon figures

In a landmark study, Navon (1977) investigated the relationship between processing at the “global” level looking at the whole, and processing at the more local level looking at the specific elements of this whole. Using arrays of stimuli where the target (e.g., a large H) was made up of many constituent elements (e.g., small squares or other letters) he found that the “global pattern is apprehended but not its components. All but three subjects did not even notice that the stimuli were made of small letters” (Navon, 1977, p. 368). In his third experiment, he looked at the effect of directing attention either to the global figure (e.g., the large H) or its constituent elements.

Inferences were made from differences in response times when the letters were conflicting (e.g., large H composed of small Ss) and when the letters were consistent (e.g., large H composed of small Hs). Navon found that participants were quicker to recognize global letters than constituent local elements. More importantly, they were also significantly impaired in recognizing local letters when they conflicted with the global letter (e.g., a large S composed of smaller Hs), but not in recognizing global letters when the images conflicted. Navon proposed that global configural aspects of an image are perceived before the local parts. This finding, which has been replicated many times, (see Kimchi, 1992 for a review) has been termed the “global precedence hypothesis.” Darling et al. (2009) found that normal participants who were the most susceptible to global interference when recognizing local letters on the Navon task performed better on a test of unfamiliar face identification. Martin and Macrae (2010) reported that individuals who show weak global interference show a reduced face inversion effect on a test of face recognition. There is therefore evidence that global processing in a Navon-style paradigm corresponds with configural processing and local processing corresponds with featural processing. Moreover, individuals with developmental prosopagnosia have been shown to have local rather than global preference (Behrmann et al., 2005) on this task. Consequently, it would be predicted that if DY has an impairment in configural processing, he should not show the global precedence effect. However, this remains an open question because Busigny et al. (2010) and Busigny and Rossion (2011) found that two different patients with acquired prosopagnosia both showed the standard global precedence effect on the Navon task.

Experiment 7 investigated the Navon effect in DY and matched controls; the latter were expected to show quicker responses in the global attention condition than in the local attention condition. The critical issues were whether DY would be slower in the global level attention condition than the local level attention condition, and whether there would be any significant difference in DY's responses at the global level (as in normal performance) in the conflicting and consistent conditions. At the local level, controls should perform more slowly in the conflicting conditions. Would, however, DY show any significant difference between response times made in the conflicting and consistent conditions?

### Stimuli

**Figure 6:**
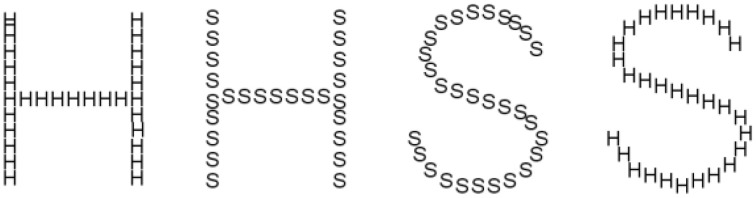
**Examples of Navon figures in two conditions, consistent (where the large figure is the same as its constituent elements) and Inconsistent (where the large figure is made up of another letter)**.

Four Navon-type letter images were created. These consisted of large figures of H and S composed of either smaller Hs or Ss, resulting in four possible images, two consistent and two conflicting (see Figure 6). The large letters were created on a template using Arial font, point size 300. The smaller letters were created using Arial font, point size 24.

### Participants

DY and nine normal controls from Experiment 3 took part in this experiment.

### Procedure

The test involved a fixation point presented for 2 s, followed by the letter image being presented on a computer screen for 100 ms using E-prime software. The image was then followed by a mask which was a simple array of dots that covered the same visual angle as the experimental stimuli. The participants' task was to respond as quickly and as accurately as possible to whether the image attended to was an H or S by pressing the H or S keys on the keyboard. The mask remained until a response was made. Each of the stimuli were presented 20 times, with a total of 80 trials; 40 of the trials were classified as “consistent” (i.e., H made of Hs and S made of Ss) and the remaining 40 trials were classified as “conflicting” (i.e., H made of Ss and S made of Hs). DY and controls carried out the test in two conditions. The first condition was to respond to the identity of the “global” letter and the second condition was to respond to the identity of the “local” letter. Accuracy and response times were recorded. Before the tests in both conditions, a series of practice items were presented using combinations of the letters L and B until the participant felt comfortable enough to proceed with the test.

### Results

In terms of accuracy, DY made 6.25% errors; one control made 23.75% errors while the error rate for the remainder ranged between 0 and 7.5% so this control was omitted from further analysis. Response times for correct responses were analyzed, and outliers that were more than 2 SDs above the mean were removed for each participant.

**Figure 7:**
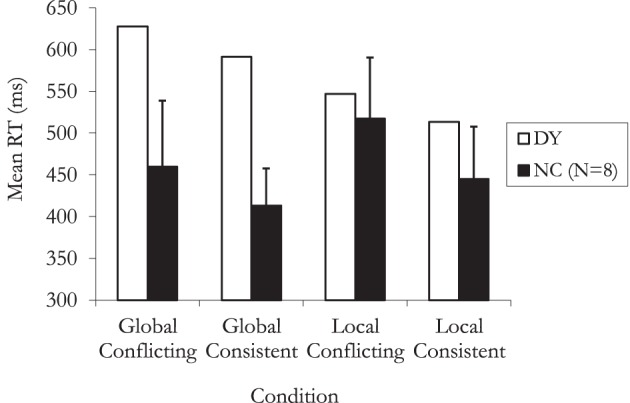
**Mean response times on the Navon task for DY and normal controls (NCs) as a function of consistency level (consistent vs. conflicting) and attention (global vs. local) (error bars represent 1 standard deviation)**.

DY's response times were found to be in the normal range in both local conditions [consistent: 513 ms, *t*_(7)_ = 1.03, ns; conflicting: 547 ms, *t*_(7)_ = 0.38, ns] (see Figure 7). However, in the global task he was significantly slower in the consistent condition [591 ms, *t*_(7)_ = 3.77, *p* = 0.007] while the difference in reaction times to that of the controls in the conflicting condition approached significance [627 ms, *t*_(7)_ = 2.01, *p* = 0.08].

The normal controls showed a classic interference effect in the local condition with the responses to the consistent letters being faster than those to the conflicting ones (local consistent *M* = 445 ms, *SD* = 62; local conflicting *M* = 517 ms, *SD* = 73), *t*_(7)_ = 3.86, *p* = 0.0062. A similar interference was found in the global condition (global consistent *M* = 413, *SD* = 44; global conflicting *M* = 460, *SD* = 79), *t*_(7)_ = 3.20, *p* = 0.015. Unlike the controls, DY was not susceptible to the interference effect in either the local condition, *t*_(69)_ = 1.39, *p* = 0.17, or the global condition, *t*_(69)_ = 1.01, *p* = 0.31.

Finally, the global and local conditions were compared to one another. For the normal controls, the global condition was faster and this difference approached significance [Global *M* = 430 ms, Local *M* = 480; *t*_(7)_ = 2.11, *p* = 0.073]. DY, on the other hand was significantly faster in the local condition (Global *M* = 609 ms, Local *M* = 530), *t*_(139)_ = 3.76, *p* < 0.001.

### Discussion

As expected, the results from the healthy controls replicated the classic Navon effect, i.e., that perception of the whole precedes that of constituent elements of an image. This is demonstrated starkly in the significant slowing down when the task is to name the constituent element when its identity conflicts with that of the global form. This happened for both the global and local conditions of the task and while Navon did not find this in his original study, the same has been found by Behrmann et al. (2005); Busigny et al. (2010) and Busigny and Rossion (2011). However, DY's response is quite abnormal and somewhat different from that of the patients studied by Busigny and colleagues. In the local task, his reaction times were comfortably within normal limits but unlike controls, he showed no interference effect. His perception of the whole is grossly abnormal however, with his reaction times being slower than that of controls in both consistent and conflicting conditions. Further, he derives no advantage when the global form matches the local elements and showed no interference effect. Finally, unlike Busigny and Rossion's (2011) patient PS, and similar to Busigny et al.'s (2010) patient GG, DY was significantly faster in the local task. This finding is consistent with intact featural processing paired with impaired global processing.

## Experiment 8: mental imagery for famous faces

Young et al. (1994) conducted a series of studies on prosopagnosic patients HJA and PH to investigate the links between visual recognition and mental imagery for faces. The results showed that it was possible for an apperceptive prosopagnosic patient such as HJA to have a profound face recognition difficulty and yet perform very well on tasks requiring him to make judgements requiring imagery for faces that he did not recognize. Experiments 8 and 9 were constructed to investigate the integrity of DY's mental imagery for faces.

### Stimuli

Following Young et al.'s (1994) procedure, four sets of 20 different people who had become famous before the onset of DY's face recognition difficulties (2001) were created. In each set, 10 had a particular feature and 10 did not. The features used were baldness (i.e., 10 people known for being balding or with shaved heads such as the actor Telly Savalas and 10 hirsute people), facial hair (10 people known for usually having mustaches or beards and 10 who were not), fair hair (10 people with fair hair and 10 with dark hair), and glasses (10 people known to usually wear spectacles and 10 who did not). Within each set, the order of the 20 names was pseudo-randomized.

### Participants

DY's performance was compared to that of eight normal controls matched for age and WTAR IQ. The controls had a mean age of 61.0 years (range 58–65) and mean IQ of 112 (range 101–117).

### Procedure

Each name within a set was presented individually and the participant was asked to imagine the person's face and answer the question relevant to that set, i.e., balding vs. not balding, facial hair vs. no facial hair, fair vs. dark hair and glasses or no glasses. Examples from each set include: balding vs. not balding, Telly Savalas (correct answer “yes”), Elvis Presley (correct answer “no”); facial hair vs. no facial hair, Groucho Marx (correct answer “yes”), Cliff Richard (correct answer “no”); fair vs. dark hair, Meg Ryan (correct answer “yes”), Jimi Hendrix (correct answer “no”); glasses vs. no glasses, Buddy Holly (correct answer “yes”), Paul McCartney (correct answer “no”).

### Results

**Figure 8:**
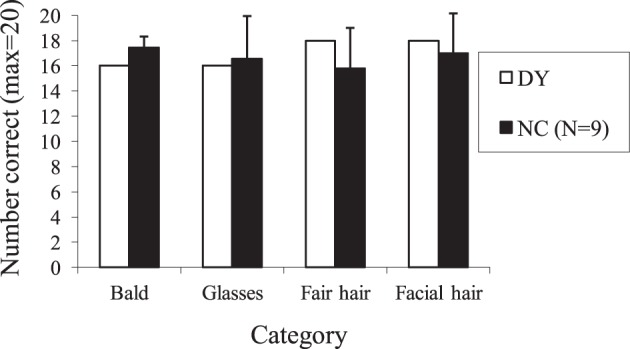
**Mean number correct on the mental imagery task for DY and normal controls (NCs) as a function of stimulus condition (error bars represent 1 standard deviation)**.

Overall, DY achieved 85% accuracy across all four categories and this matched the average of the control participants which was also 85%. From Figure 8 it can be seen that across the four categories, DY's performance was at the mean level of the controls or was within one standard deviation. As a result, no further analysis was conducted.

## Experiment 9: mental imagery free recall

Experiment 8 involved a simple “yes/no” decision and DY's performance seemed perfectly intact. However, it could be argued that good performance on this task is driven by propositional knowledge of different attributes of individuals' faces and that this is not a convincing demonstration of the intactness of DY's internal representations of faces. Therefore, Experiment 9 examined DY's mental representations by conducting a free recall mental imagery task in which he was asked to describe in his own words what a number of people looked like.

### Stimuli and procedure

The names of 10 famous personalities were read out to participants one at a time. They were asked to describe the person's face to their best of their ability and to avoid semantic attributes, i.e., to base the descriptions purely on visual features. The protocols of the descriptions were transcribed and the resulting transcripts had any remaining non-visual semantic information removed. The 10 verbal descriptions produced by each participant were then given to a set of six raters along with the 10 target names. The raters were simply asked to match the descriptions to the names. From this procedure, the dependent variable for each experimental participant was the average number of their descriptions that were correctly matched to target names by the raters.

### Participants

DY's performance was compared to that of eight normal controls matched for age and WTAR IQ. The controls had a mean age of 61.2 (range 51–69) and mean IQ of 107.4 (range 90–117).

### Results

Across the six raters, DY's verbal descriptions scored 8/10 which was well-within the range of the normal controls (*M* = 8.4, *SD* = 1.16), *t*_(7)_ = 0.33, *p* > 0.05.

### Discussion of experiments 8 and 9

The results from Experiments 8 and 9 show that, despite DY having profound problems in recognizing faces, he nonetheless is able to make very good judgments and provide recognizable descriptions from his internal mental images of famous people's faces. His performance (in Experiment 8) is similar to that of the apperceptive prosopagnosic patient HJA who also performed normally on facial imagery tasks for faces that he could not recognize. The mental imagery studies strongly imply that DY's internal representations of the faces of famous people are largely intact. We conclude that his face recognition units are preserved and can be accessed from familiar names and from the semantic system (see Craigie and Hanley, 1993, 1997, for discussion of how this form of retrieval appears to take place).

## General discussion

In this study, we have presented data from a patient who, in the context of relatively unimpaired naming of familiar objects, complains of profound face recognition difficulties. His impairment with once familiar faces is so severe that, in everyday life, he is unable to recognize even close members of his family such as his wife, children, or grandchildren. Table 4 presents a summary of DY's performance as standardized scores relative to controls on the main background tests and the nine experimental studies. Table 4 also indicates the type of processing that was under investigation in each experiment.

**Table 4:** **Summary of DY's performance on background and experimental tests relative to controls with z-scores where possible (numbers in brackets denote experiment numbers)**.

**Test/Expt**	**Sub-test/Cond**	**Measure**	**Testing**	**DY**	**NC_$\dot{x}$_**	**NC_sd_**	**z-score**	**Interpretation**
Mooney faces		Acc (max = 160)	Holistic processing	127	146.4	7.2	2.69	Impaired
		RT	”	1317	940	117	3.21	Impaired
BFMT		Acc	Face Perception	41				Normal
		RT						Impaired
Within-category (1)	Flags	Acc (max = 20)	Object naming	11	14.25	2.9	1.1	Normal
	Cars	”	”	10	13.25	4.7	0.7	Normal
	Football shirts	”	”	14	15.12	2.40	0.47	Normal
	Buildings	”	”	15	15.3	3.08	0.10	Normal
Essex-Exeter (2)			Object and Face naming	35	41.2	6.50	0.95	Normal
CFMT (3)	Upright	Acc (max = 72)	Configural processing	26	46.9	6.26	3.34	Impaired
	Inverted	Acc (max = 72)	Featural processing	31	35.2	5.43	0.77	Normal
		Inversion effect	Configural processing	−0.088	0.1431	0.01	33.1	Impaired
Face fracturing (4)	Intact	Acc (max = 25)	Featural processing	23	24	0.63	1.58	Normal
	Fractured	”	”	22	23.17	1.17	1.00	Normal
	Intact	RT	Featural processing	11,551	2574	327	−27.4	Impaired
	Fractured	”	”	7585	5214	600	−3.95	Impaired
Inverted famous faces (5)		Acc (max = 25)	Featural processing	10	13.1	5.00	0.62	Normal
		RT	”	3586	3120	1054	−0.44	Normal
External features of faces (6)		Acc (max = 42)	Featural processing	31	32.1	6.37	0.17	Normal
Navon task (7)		RT	Configural processing					Impaired
Mental imagery (8)	Bald	Acc (max = 20)	Face Recognition Units	16	17.4	0.88	1.64	Normal
	Glasses	”	”	16	16.6	3.40	0.16	Normal
	Fair hair	”	”	18	15.8	3.23	−0.69	Normal
	Facial hair	”	”	18	17	3.16	−0.32	Normal
FR mental imagery (9)		Acc (max = 10)	Face Recognition Units	8	8.4	1.16	0.34	Normal

*NC_$\dot{x}$_, Normal Control (NC) mean; NC_sd_, NC standard deviation; BFMT, Benton Face Matching Task; Essex-Exeter, Essex Exeter Matched Difficulty Task; CFMT, Cambridge Face Memory Task; RT, mean response time (ms); Acc, Accuracy; FR, Free Recall*.

Consistent with his reported problems, DY performed much worse than controls at learning new upright faces (Experiment 3). Paradoxically, he identified famous faces more accurately than might have been expected (Experiment 2) and his accuracy in unfamiliar face-matching tasks (e.g., Benton and van Allen, 1968) put him within the “normal” range. However, closer inspection of his performance revealed that his higher-than-expected accuracy was based almost entirely on identification of particular features within faces. Mistaking a photo of one of the researchers for George Michael because the former has a goatee beard provided an example of how reliant he is on recognition of individual features. This featural strategy revealed itself clearly when he took much longer to identify upright famous faces (Experiment 4) than controls. Similarly the time that he took to complete the Benton unfamiliar face-matching test was grossly abnormal, and his verbal protocols clearly revealed a laborious feature-by-feature matching strategy.

The results of subsequent experiments provided further evidence of DY's reliance on featural processing. He produced normal or relatively preserved performance on tests of familiar face recognition that depend on processing of featural information such as recognition of fractured familiar faces (Experiment 4), recognition of inverted familiar faces (Experiment 5), and recognition of famous faces from their external features (Experiment 6).

In none of these tasks is configural information readily available from faces, and DY appears to be relatively unaffected by its absence. DY's performance in these four experiments, together with his unimpaired ability to name objects, represents a double dissociation with the object agnosic patient CK (Moscovitch et al., 1997; Moscovitch and Moscovitch, 2000) who performed badly at object recognition and on face recognition tasks that require featural processing despite excellent recognition of faces when configural information is available.

When configural information must be used to achieve normal levels of performance, as in the time required to recognize upright famous faces, DY performed much worse than controls (Experiment 4). Consistent with a holistic processing deficit, DY performed poorly on the Mooney faces. Consistent with a more general global processing deficit, DY performed differently from controls in Experiment 7 where he showed evidence of impaired processing of the global form of the Navon Figures. In line with the views of Farah and Moscovitch, therefore, the performance of DY provides strong evidence of a patient with prosopagnosia whose problems in recognizing familiar faces is the consequence of a holistic/configural processing deficit.

Nevertheless, DY's accurate recognition of upright familiar faces (Experiment 2) raises an important question. Why is he able to identify so many famous faces via a featural processing strategy when the performance of many prosopagnosics on such tasks is either at chance or is severely impaired? Is this because DY has unusually good featural processing skills? Some prosopagnosics such as HJA and MS (Newcombe et al., 1989) do suffer from object recognition deficits as well as from face recognition deficits. So, their total inability to identify any familiar faces probably does reflect severe featural as well as configural processing impairments.

However, the situation is different with other prosopagnosic patients whose accuracy at familiar face recognition is severely impaired such as LH (Levine and Calvanio, 1989; Farah et al., 1995), FB (Riddoch et al., 2008), and WJ (McNeil and Warrington, 1993), All three cases appear to have preserved featural processing: LH was able to recognize difficult objects well and performed well at matching inverted unfamiliar faces; FB showed excellent ability to name familiar objects and to learn names for greebles (complex novel shapes); WJ showed excellent ability to identify sheep facesIt therefore seems unlikely that the featural skills of DY are markedly superior to those of all three of these patients. So why are they much less accurate than DY at familiar face identification? One possible explanation is that familiar face identification problems in these three patients reflect a more associative form of prosopagnosia than that experienced by DY. In these three individuals, there may be an impairment either to the face recognition units themselves or to the connections between the face recognition units and the rest of the cognitive system (Burton and Young, 1999). A problem of this kind would impair identification of familiar faces even if featural processing was entirely preserved.

It is also interesting to note that DY's ability to identify inverted and fractured familiar faces makes a striking contrast with the performance of patient DC, reported by Rivest et al. (2009). Like DY, DC had excellent object recognition skills, consistent with preserved featural processing despite problems in identifying familiar faces and matching unfamiliar faces. Unlike DY, however DC, was impaired relative to controls at identifying fractured and inverted familiar faces. For example, DC recognized only 9.1% of inverted pictures of famous faces that he could identify when presented upright. The corresponding figure for controls was 52%. Rivest et al. concluded that the parts-based system cannot *by itself* identify familiar faces and that the configural processing system must interact with the featural system to recognize fractured and inverted faces. A configural processing deficit, they argue, will invariably lead to a problem in identifying inverted faces. They therefore predict that it should not be possible to observe a prosopagnosic patient who provides a double dissociation with the object agnosic CK by performing well at object recognition and at the recognition of inverted and fractured faces. As we suggested earlier, however, DY appears to represent exactly such a case. It is therefore worth considering instead whether an impairment at the level of the face recognition units (Burton and Young, 1999) might be able to explain DC's poor performance when recognizing familiar faces. Because a face recognition unit impairment would affect face processing at a point at which featural and configural processing have already been completed, it would disrupt identification of familiar faces regardless of whether they were upright, inverted or fractured. This is precisely the pattern of performance that Rivest et al. observed in DC.

In conclusion, although there is considerable evidence that prosopagnosics' impaired configural processing interferes with their processing of unfamiliar faces (e.g., Rossion et al., 2011), there is much less evidence that a configural processing deficit is the cause of impaired identification of familiar faces in prosopagnosia. Indeed, it appears that the familiar face processing problems experienced by prosopagnosic patients such as DC (Rivest et al., 2009), LH (Levine and Calvanio, 1989; Farah et al., 1995), FB (Riddoch et al., 2008), and WJ (McNeil and Warrington, 1993) can be readily explained in terms of a problem at the level of the face recognition units (Burton and Young, 1999). In cases of apperceptive prosopagnosia such as HJA or ME (Young et al., 1994), there is evidence of impaired featural as well as impaired configural processing. The case of DY is therefore unusual in that he has no problem in recognizing objects (consistent with unimpaired featural processing). Neither does he have a problem in identifying inverted or fractured familiar faces or in accessing mental images of familiar faces (consistent with unimpaired face recognition units). He therefore presents the clearest case yet reported of an acquired prosopagnosic whose impaired processing of familiar faces appears to be the consequence of a configural processing deficit.

### Conflict of interest statement

The authors declare that the research was conducted in the absence of any commercial or financial relationships that could be construed as a potential conflict of interest.

## Footnotes

^1^Two examples of DY's face recognition problems are presented in the Supplementary Material. DY was asked if he knew the name of the celebrity and if he provided a name, to give a rating from 1 to 10 of his confidence.
